# Comparative Assessment of Regular and Persulfate Oxidative Foams in Air Sparging for Trichloroethylene Dense Nonaqueous Phase Liquid Remediation

**DOI:** 10.1021/acsenvironau.5c00046

**Published:** 2025-06-25

**Authors:** Xuyen Thi Hong Luong, Chenju Liang

**Affiliations:** Department of Environmental Engineering, 34916 National Chung Hsing University, 145 Xingda Road, Taichung 402, Taiwan

**Keywords:** Air Sparging (AS), Oxidative Foam, Sodium Persulfate (SPS), Surfactant, Trichloroethylene (TCE), Dense Nonaqueous Phase Liquid (DNAPL)

## Abstract

This study investigated the feasibility of foam-enhanced air sparging (FEAS) for remediating trichloroethylene (TCE) dense nonaqueous phase liquid (DNAPL) in water. Various surfactants, including polyoxyethylene (20) sorbitan monooleate (TW80), sodium dodecyl sulfate (SDS), sodium α-olefin sulfonate (AOS), and TW80/SDS and TW80/AOS combinations, were used to generate foam, which were evaluated for foam stability and quality. AOS (32 mM) exhibited the highest foam stability (∼345 min) and quality (∼99.6%) under controlled conditions. Phase contrast microscopy analysis showed foam sizes of 290–400 μm with thin film thicknesses of 6–9 μm. FEAS was tested with and without sodium persulfate (SPS) oxidant (oxidative foam) to treat approximately 10 g of TCE DNAPL in 1 L of water. Injecting AOS foam (32 mM) or oxidative foam AOS (32 mM)/SPS (50 or 1700 mM) for 2 h dissolved 60–82% of TCE, compared to only 4–7% with N_2_ injection. The surfactant-stabilized interface in foam facilitated TCE adsorption, increasing its partitioning into bubbles, leading to enhanced volatilization. In the lamella region, surfactant layers promoted TCE dissolution, while SPS aided its mineralization. With oxidative foam at a higher SPS concentration (1700 mM) and an extended reaction time (240 h), TCE mineralization increased to 40–74% across different foam injection rates. These results highlight oxidative FEAS as a promising improvement over conventional air sparging, significantly enhancing TCE dissolution, volatilization, and oxidation.

## Graphical abstract

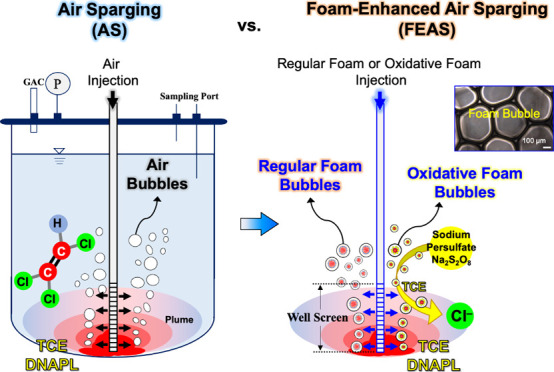

## 1 Introduction

Soil and groundwater contamination remain significant environmental challenges, particularly in cases involving dense nonaqueous phase liquids (DNAPLs), which are notoriously difficult to remediate. Trichloroethylene (TCE), which is a commonly used industrial solvent, is a prominent example. Due to its low water solubility and complex subsurface migration behavior,^1,2^ accidental or improper releases of TCE often lead to the formation of persistent DNAPL source zones. Even small quantities can act as long-term sources of dissolved contamination, significantly extending remediation timelines, often over decades.^3^ Given the persistence of TCE DNAPL and the stringent regulatory standards established to protect human health,^1,2^ the development of effective subsurface remediation technologies remains a critical environmental priority.

Air sparging (AS) is a widely applied in situ remediation technique that promotes the volatilization and biodegradation of contaminants in the saturated zone.^4–6^ In this process, air bubbles injected into the subsurface enhance mass transfer of volatile contaminants, which are subsequently transported to the unsaturated zone and extracted using a soil vapor extraction (SVE) system.^7^ However, the treatment of TCE DNAPL using AS is particularly challenging, primarily due to TCE’s low water solubility (∼1100 mg/L at 20 °C).^8^ Moreover, the success of AS depends heavily on accurately placing injection wells near DNAPL source zones, a task complicated by the heterogeneity and complexity of site conditions.^6,9^

Couto et al.^10^ investigated the remediation of diesel-contaminated sandy soils using surfactant solutions, regular foams, and colloidal gas aphrons as treatment fluids. Their findings showed that, after injecting four pore volumes of surfactant solution, both gas aphrons and regular foams achieved significantly higher removal efficiencies compared with the surfactant solution alone. Jeong et al.^11^ investigated the use of surfactant foam flooding to enhance the displacement of residual TCE in a micromodel (59 × 42 × 0.13 mm) and quantified its removal efficiency across different porous media using an image analyzer. Their findings demonstrated that surfactant foam flooding was more effective in mobilizing residual TCE compared to conventional remediation methods, such as gas and water flooding or surfactant flushing. This increased efficiency was attributed to the reduction of the interfacial tension between the contaminant and the aqueous phase. The results suggest that surfactant foam is particularly advantageous over surfactant solution flushing, especially in low-permeability zones.^12^

Reducing the amount of surfactant injected into the subsurface, while maximizing DNAPL removal, remains an important area of research. Among four main types of surfactants, zwitterionic and cationic surfactants are generally less suitable for remediation due to their electrostatic attraction to negatively charged soils.^13^ In contrast, anionic surfactants, which carry a negatively charged headgroup, exhibit lower soil adsorption compared to cationic and zwitterionic surfactants. Nonionic surfactants, on the other hand, significantly enhance the solubility of organic compounds and improve system stability by reducing the sensitivity to water hardness. To minimize surfactant loss in surfactant-enhanced aquifer remediation applications, a combination of anionic and nonionic surfactants is often preferred.^14^ In this study, polyoxyethylene (20) sorbitan monooleate (TW80) was selected as a representative of nonionic surfactant, while sodium docecyl sulfate (SDS) and sodium α-olefin sulfonate (AOS) were chosen as the representatives of anionic surfactants for foam generation. Moreover, TW80 was selected as a representative nonionic surfactant due to its low critical micelle concentration (CMC) of 0.012 mM,^15^ which promotes micelle formation and enhances the solubilization of hydrophobic organic contaminants—an important factor for improving desorption from the aquifer soil matrix.^16^ Its low affinity for soil also minimizes surfactant loss through adsorption. TW80 is well-established and widely used in various environmental remediation applications.^7^ SDS and AOS were chosen as representative anionic surfactants primarily for their strong foam-generation capabilities, which are critical for effective mobility control. SDS also demonstrates excellent detergency, facilitating the release of contaminants from soil particles.^10^ AOS provides the additional benefit of high tolerance to hard water, maintaining foam stability and detergency in aquifers with elevated mineral content.^17^

If TCE DNAPL is solubilized using a surfactant foam with a reduced surfactant dosage, the dissolved TCE may experience increased contact with the oxidant, enhancing its potential for oxidative degradation. Consequently, the integration of a chemical oxidant with surfactant foam presents a more comprehensive and efficient remediation strategy. Sodium persulfate (Na_2_S_2_O_8_, SPS) has been identified as a promising oxidant for degrading DNAPL pollutants. When activated, SPS generates sulfate radical (SO_4_^•–^), which can effectively degrade a wide range of contaminants.^18,19^ By injection of surfactant foam into the subsurface, DNAPL pollutants can be more effectively solubilized, enhancing their dissolution and increasing the interaction between SO_4_^•–^ and contaminants. This, in turn, improves the efficiency of the subsequent oxidative degradation process.

Liang and Yang^7^ evaluated the effectiveness of TW80/SPS oxidative foam in removing diesel from vadose soils, comparing it to SVE alone. Their results demonstrated a significantly higher removal rate of 37% after 10 h of TW80/SPS foam flushing compared to just 3% with nitrogen gas flushing alone. Moreover, Bajagain et al.^20^ investigated the use of foam containing SPS for the oxidation of total petroleum hydrocarbons (TPHs) in contaminated soils. Their study involved spraying SPS-infused foam onto the surface of TPH-contaminated soils, resulting in approximately 80% degradation of TPHs, compared to 52% removal with regular foam alone. These findings highlight the role of SPS in enhancing the oxidative activity of foam, significantly improving the contaminant removal efficiency for in situ environmental remediation. Therefore, this research aimed to investigate the potential of using regular foam and SPS oxidative foam for remediating TCE DNAPL contamination as an enhanced AS technology. The study was conducted in two phases. First, the characteristics of various regular foams (TW80, SDS, AOS, TW80/SDS, and TW80/AOS) were analyzed to determine the suitable formulation. This selected foam was then further modified to produce an SPS oxidative foam. Second, the feasibility of foam-enhanced air sparging (FEAS), with and without the addition of SPS as an oxidant, was evaluated for its effectiveness in treating TCE DNAPL. The study also examined the mass distribution of TCE among dissolution, volatilization, and oxidation processes in a laboratory-scale reactor in the aqueous phase.

## 2 Materials and Methods

### 2.1 Chemicals and Materials

Trichloroethylene (C_2_HCl_3_, ≥99.9%), sodium dodecyl sulfate (CH_3_(CH_2_)_11_OSO_3_Na, ≥95%), and poly oxyethylene (20) sorbitan monooleate (C_24_H_46_O_5_(C_2_H_4_O)_20_OH, 99%) were purchased from Sigma-Aldrich. Sodium α-olefin sulfonate (C_14_–C_16_, with industrial purity and used as received without any further purification) was purchased from King Yu Chemicals Co., Ltd., Taiwan. Acetonitrile (C_2_H_3_N, >99.9%) was purchased from Aencore Chemical PTY., Ltd. Sodium persulfate (Na_2_S_2_O_8_, ≥99%) was purchased from Merch, Germany. Sodium nitrate (NaNO_3_, ≥99%) and potassium iodide (KI, ≥99.7%) were purchased from Union Chemical Works. Sodium chloride (NaCl, ≥99.5%) was purchased from Fluka, Germany. Sodium bicarbonate (NaHCO_3_, ≥99.7%) was purchased from Riedel-de Haën, Germany. Methanol (CH_3_OH, ≥99.9%) was purchased from Macron Fine Chemical, USA. *n*-Hexane (C_6_H_14_, ≥ 95.0%) was obtained from Tedia, USA. Granular activated carbon (GAC) was purchased from Li Jing Viscard Co. Ltd., Taiwan. Water was purified by using a reverse osmosis (RO) purification system (Model XL-300 A, Sky water Ltd., Taiwan).

### 2.2 Experimental Procedure

In the initial phase, the properties of regular foams (TW80, SDS, AOS, TW80/SDS, TW80/AOS) and oxidative foams (individual surfactants combined with SPS oxidant), including foam quality and stability, were evaluated based on the procedure outlined by Liang and Yang.^7^ These evaluations were conducted to identify a suitable surfactant candidate for further investigation in FEAS experiments. The primary objective of this study was to investigate the fundamental interactions and treatment mechanisms between injected regular/oxidative foams and TCE DNAPL under controlled, well-defined conditions. Aqueous batch experiments were conducted to examine the FEAS treatment of TCE DNAPL, allowing for precise control of key variables and providing a foundation for future soil column or field-scale investigations. Subsequently, in the second phase, batch experiments and examinations of regular foam and oxidative foam (mixed with different SPS concentrations) were conducted for enhanced treatment of TCE DNAPL. The control tests with only N_2_ injection, addition of surfactant alone, or mixing with SPS and surfactant together were also conducted in parallel for comparison of removal efficiency of TCE DNAPL. Figure 1 depicts the schematic diagram of the experimental setup for the second phase batch experiment. The experimental design flowchart, including detailed parameters, is shown in Figure S1.

**Figure 1:**
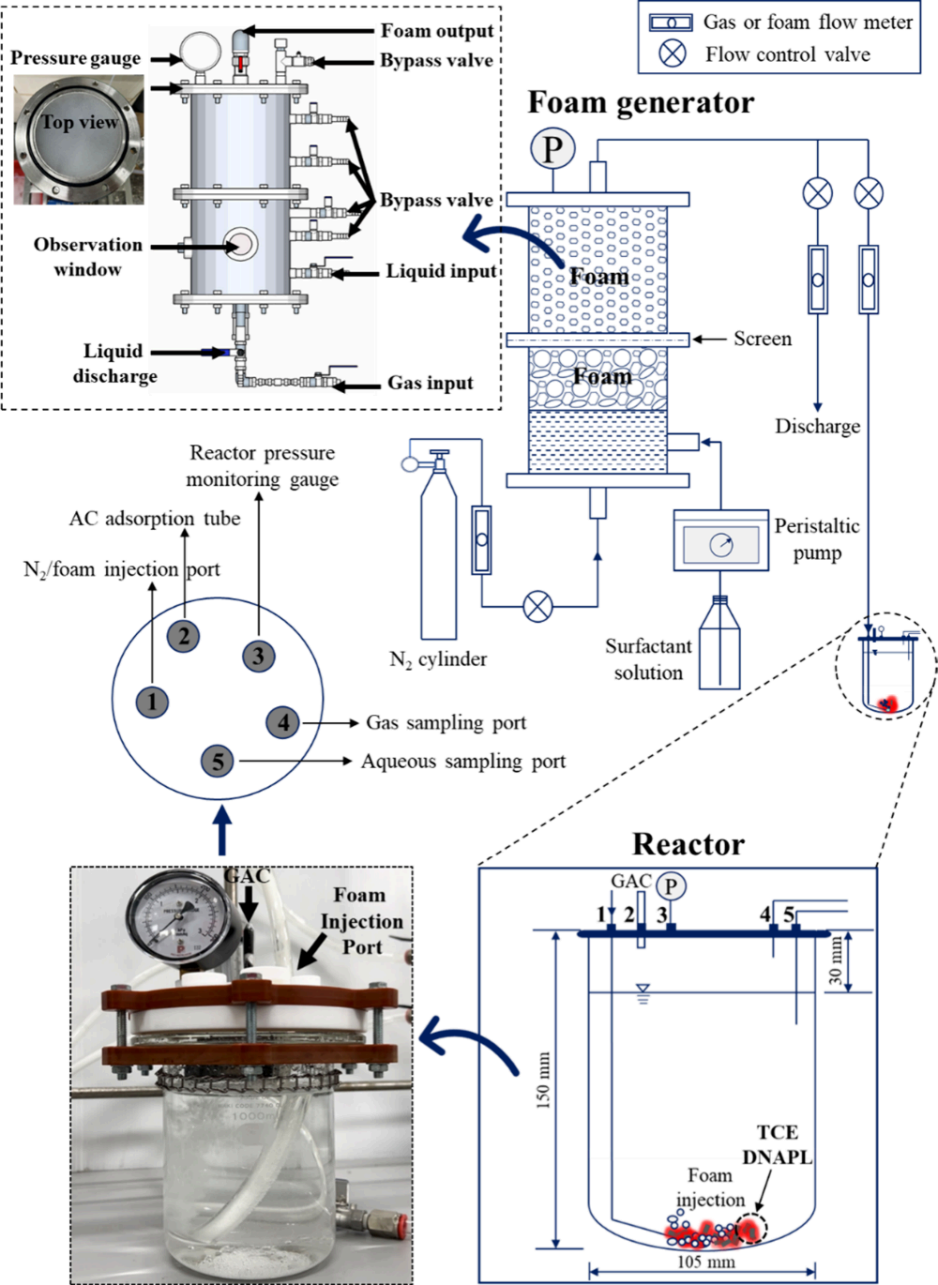
Illustration of the FEAS experimental setup.

The experimental setup included a cylinder supplying N_2_ gas, which passes through a stainless-steel foam generator. Once foam generation became continuous, the outflow valve of the foam generator was opened, allowing the foam to flow into a 1.3 L heavy-wall plain pressure reaction flask (glass reactor) at room temperature. The reactor was sealed with a Teflon cap equipped with multiple ports. An activated carbon tube (ORBO sorbent tubes, Supelco) for adsorbing volatilized vapor and a foam injection pipeline were inserted through Teflon-lined septum ports. A pressure gauge was installed, and two additional ports were designated for withdrawing aqueous and gaseous samples. It should be noted that, to minimize potential confounding effects of O_2_, inert N_2_ gas was injected instead of air for foam generation, preventing aerobic activity induction. The foam was generated in the foam generator with a fixed liquid-to-gas ratio, consisting of 2 L of foaming surfactant and a N_2_ inflow rate of 2 L min^–1^.^7^ A peristaltic pump delivered the foaming surfactant, ensuring a consistent liquid-to-gas ratio. During foam injection into the reactor, the internal pressure of the foam generator was regulated below 1.5 kg cm^–2^ by using a pressure release valve.

The reactor was filled with 1 L of water, and 7.5 mL of TCE DNAPL (10,950 mg) was injected at the bottom to simulate a significant DNAPL source. This mass, approximately ten times the aqueous solubility of TCE (∼1,100 mg/L at 25 °C), was selected to represent a scenario with residual and potentially pooled DNAPL, leading to environmentally relevant contamination and allowing for evaluation of remediation performance under challenging conditions. A headspace of approximately 3 cm in height was present within the reactor. TCE trapped in the GAC adsorption tube during N_2_ flow or foam injection was analyzed to determine the mass of volatilized TCE venting through the tube. Throughout each experiment, the total volume of aqueous samples collected for analysis at various sampling periods was below 10% of the total liquid volume (1 L).

For each experimental condition, for example, when testing AOS surfactant at five different concentration levels, foam was collected by using a 100 mL graduated cylinder. As a result, a set of five 100 mL cylinders was used to collect samples corresponding to the five concentrations. Each condition was tested in triplicate, and the data presented represent the average of three measurements obtained from the 100 mL of foam samples collected during each replicate run. A consistent sample volume of 100 mL was used for characterization in all of the experimental runs. Foam stability was assessed by measuring the time required for the foam to disintegrate to half of its original volume, reflecting its resistance to bubble breakdown. Foam quality (η, %) was determined as the percentage of gas content in the total foam volume, as calculated using the following eq 1:

$$\eta \;\left(\%\right)=\frac{Gas\;volume\;(V_{g})\;\left(mL\right)}{Total\;foam\;volume\;\left(V_{f}\right)\;\left(mL\right)}\times 100\%$$ 1

where gas volume (*V*_g_, mL) = total foam volume (*V*_f_, mL) – water volume (mL).

Upon completion of the FEAS experiments, the upper portion of the remaining TCE dissolved in the aqueous phase (*V*_aq_) was collected at a flow rate of 20 mL min^–1^ by using a peristaltic pump for analysis. Subsequently, approximately 150 mL of solution (*V*_DNAPL_) remained at the bottom of the reactor containing residual TCE DNAPL. This solution was extracted with hexane to determine the total mass of TCE remaining in the reactor. The overall mass of TCE analyzed and recovered (*M*_DNAPL_) in the gaseous, aqueous, and DNAPL phases was calculated using the following eq 2:

$$M_{\mathrm{DNAPL}}=C_{\mathrm{GAC},\mathrm{ext}}\times V_{\mathrm{GAC},\mathrm{ext}}+C_{\mathrm{aq}}\times V_{\mathrm{aq}}+C_{\mathrm{DNAPL},\mathrm{ext}}\times V_{\mathrm{DNAPL}}$$ 2

where *C*_GAC,ext_ is the TCE concentration in the GAC extract and *V*_GAC,ext_ is the volume of extract used for desorbing TCE from GAC. *C*_aq_ represents the aqueous TCE concentration in solution (*V*_aq_), while *C*_DNAPL,ext_ is the TCE concentration in the extract of the DNAPL-containing solution (*V*_DNAPL_).

### 2.3 Analytical Methods

The pH (HANNA HI 1131B pH combination electrode), oxidation reduction potential (ORP) (METTLER TOLEDO Inlab redox combination electrode), and chloride ions (combination electrode chloride 9617B Orion) were measured using a pH/ISE meter (HANNA Instruments HI 5222). Dissolved oxygen (DO) was measured by a WTW Oxi 3310 portable DO meter with a CellOx 325 Probe. The surface tension (SFT) and contact angle (CA) were measured by using a Drop Shape Analyzer (DSA25, KRÜSS). Foam images were captured using a Nikon SMZ800N stereo microscope (bright-field (BF) microscope) and a Leica DMI4000 B fluorescent microscope (phase-contrast (PH) microscope). SPS was measured using a spectrophotometric method^21^ with a UV–vis spectrophotometer (HACH DR/2400) at 400 nm. TCE DNAPL and GAC sorbed TCE were extracted using hexane, and gaseous TCE sample was withdrawn using a gastight syringe (VICI gas syringe series A-2, USA) for analysis. The TCE extract and gaseous TCE were analyzed using a gas chromatograph (GC, Agilent 7890 A) with an Agilent J&W Scientific DB-624 column (60 m × 0.25 mm × 1.4 μm) and equipped with a mass spectrometer (MS, Agilent 5975 C) in electron impact mode in accordance with the analytical method for volatile organic compounds (VOCs) established by the Taiwan National Institute of Environmental Analysis (NIEA Method W785-54 B). Aqueous TCE samples were filtered (0.2 μm polytetrafluorethylene filter, Advantec, KS-13, placed within a stainless syringe holder) and then measured using a high-performance liquid chromatography system (HPLC, PerkinElmer Fexar LC) equipped with a photodiode array (PDA) detector. A Brownlee SPP C18 column (4.6 × 150 mm, particle size 2.7 μm) was used for analyte separation. An HPLC/PDA detector was set to detect TCE at a wavelength of 210 nm. Acetonitrile and RO water, mixed at a ratio 70/30 (v/v), was used as the HPLC/PDA mobile phase with a flow rate of 1.0 mL min^–1^.

## 3 Results and Discussion

### 3.1 Characteristics of Regular and Oxidative Foam

Foam characteristics were assessed using 100 mL graduated cylinders after generation from the surfactant solution, as illustrated in Figure 2a. Figure 2b,c shows the foam stability and quality as functions of varying concentrations of individual surfactant (AOS, SDS, and TW80) and surfactant combinations (TW80/AOS and TW80/SDS), respectively. The underlying raw data for these results are presented in Table S2. It can be seen that the foam stability increased with the rising concentration of each surfactant under consistent experimental conditions (N_2_/liquid ratio of 2.0/2.0 (L min^–1^)/L and screen #60 mesh, 0.25 mm in diameter). Particularly, when the surfactant concentration exceeded four times the critical micelle concentration (CMC), the foam stability of AOS and SDS gradually increased, eventually reaching a steady state of approximately 345 min for AOS and 240 min for SDS, respectively, while the TW80 concentration exceeded 800 times the CMC and the foam stability reached 150 min. Zheng et al. reported that maximum foam stability of AOS reached approximately 300 min at concentrations from 15 to 20 mM.^22^ Similarly, Liang and Yang demonstrated that TW80 achieved foam stability of approximately 120 min when its concentration exceeded 8 mM, with no significant improvement at higher concentrations.^7^ These results indicate that foam stability generally increases with rising surfactant concentration until a plateau is reached.^7,23^ Contact angle measurements of surfactants on quartz glass at various concentrations (Table S2) showed a consistent decrease in CA with increasing concentration: AOS (2.0–32.0 mM, CA: 24.9–9.7°), SDS (16.0–256.0 mM, CA: 19.8–9.4°), and TW80 (2.4–31.2 mM, CA: 29.9–12.3°). This trend indicates that higher surfactant concentrations improve wetting by lowering the CA. As concentration increases, foam stability typically improves while CA decreases.^24,25^ A lower CA reflects enhanced surfactant alignment at the air–water interface, reducing interfacial energy. However, further increases in concentration offer limited benefit, as wetting efficiency reaches a plateau.^26^ Among the three studied surfactants, AOS exhibited the highest foam stability, maintaining stability for approximately 345 min at concentrations above 16 mM. As shown in Figure 2b and Table S2, the foam quality of AOS, SDS, and TW80 remained consistently high, ranging from 96.9% to 99.7%. The results indicate that over 96% of the foam volume was composed of gas for all individual surfactant foam, with AOS exhibiting the highest foam quality at 99.6%.

**Figure 2:**
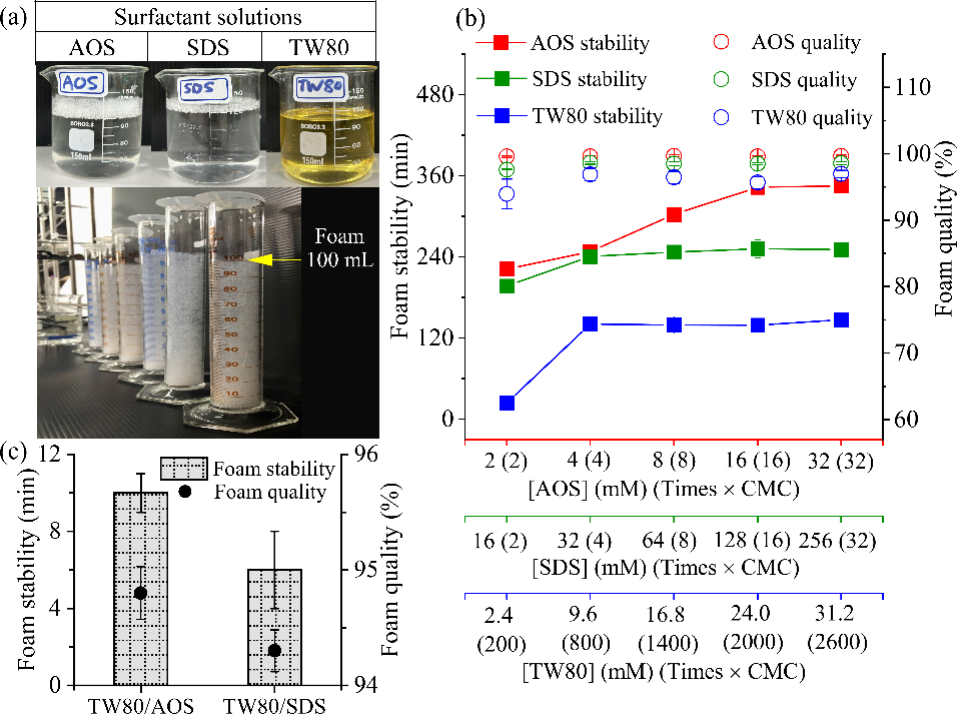
Characteristics of foams generated using different concentrations of individual surfactants (AOS, SDS, and TW80) and blended surfactants (TW80/AOS at 16.8/32.0 mM and TW80/SDS at 16.8 mM/128.0 mM). (a) Images of surfactant solutions and the generated foam, along with foam stability and quality assessments for (b) individual surfactants and (c) blended surfactants.

Additionally, an example image of the AOS foam was captured immediately after its formation. As shown in Figure 3, the AOS foam comprises a continuous liquid phase and a dispersed gas phase. The consistent halo ring surrounding the gas phase offers partial evidence of phase transitions between the liquid and gas phases. Notably, the gas dispersion creates a complex structure, in which bubbles are separated by thin liquid layers, known as lamellae.^12^ These lamellae may either extend through pores or break under the resistance of water flow. However, the AOS foam image reveals the formation of a plateau border where three foam bubbles converge, with each angle at the boundary measuring approximately 120°, indicating that the AOS foam produced under the given generation conditions may represent an idealized foam.^27^ As shown in Figure 2b, foam stability for each surfactant reaches a plateau at concentrations above the CMC. While near-optimal foam stability and quality are achieved at the onset of this plateau, the selection of surfactant concentrations for further testing was based on more than just meeting this minimum threshold. A higher concentration within the plateau was chosen to promote the formation of “idealized foam” structures, characterized by distinct Plateau borders forming angles close to 120° at bubble junctions. These structures, illustrated for AOS in Figure 3 and visually confirmed for SDS and TW80 in Figure S3c,e, are indicative of more robust and stable foam films (lamellae). It is hypothesized that maximizing the presence of these idealized structures enhances the foam consistency and performance during subsequent treatment experiments. Therefore, the following concentrations were selected for continued investigation: 32 mM AOS (32× CMC), 128 mM SDS (16× CMC), and 16.8 mM TW80 (1400× CMC).

**Figure 3:**
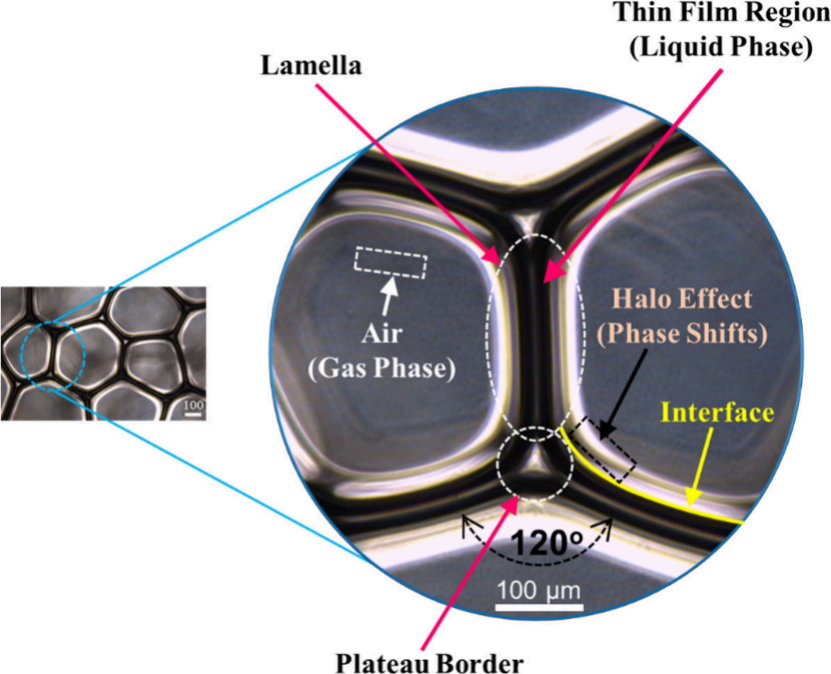
AOS foam structure captured using a phase-contrast (PH) microscope under foam generation conditions: #60 mesh screen and N_2_/liquid ratio of 2.0/2.0 (L min^–1^)/L.

To evaluate the differences in foam characteristics between blended nonionic and anionic surfactant combinations and foams generated from individual surfactants (as shown in Figure 2c and Table S2), both TW80/AOS (16.8 mM/32.0 mM) and TW80/SDS (16.8 mM/128.0 mM) foams exhibited low foam quality (∼94–95%) and limited foam stability (∼6–10 min). Additionally, the measured CAs for TW80/AOS (16.8 mM/32.0 mM) and TW80/SDS (16.8 mM/128.0 mM) mixtures were 17.4° ± 2.4 and 17.1° ± 3.4, respectively. These relatively higher values, compared to those of individual surfactants at higher concentrations, suggest reduced wetting efficiency. As such, these anionic/nonionic mixtures may not be optimal for further investigation in this study. Xu et al.^28^ reported that foams produced from anionic/nonionic surfactant mixtures exhibited optimal stability under elevated temperature (50–60 °C) and pressure conditions (∼4 MPa). These results indicate that the current operational parameters of the foam generator at room temperature may not be ideal for producing stable foam with anionic and nonionic surfactant combinations in this study. Similarly, Huang et al.^29^ investigated the effect of the molar ratio of Triton X-100 (nonionic) to SDS (anionic) on foam stability. They found that, when the molar ratio was less than 0.2, the stability of Triton X-100/SDS foam was under 20 min. They further suggested that a molar ratio exceeding 0.5 is necessary to achieve maximum foam stability for anionic SDS surfactants combined with nonionic surfactants. In this study, the molar ratio used ranged from 0.13 to 0.52, which appears to be below the recommended optimal condition. Therefore, further evaluation of the foam generation conditions is needed to optimize foam characteristics for specific environmental applications when blended surfactants are used. To fully harness the potential of surfactant blends, particularly their cost-efficiency and synergistic performance in applications such as FEAS, a comprehensive evaluation is warranted. This should include optimization of key parameters such as the molar ratio between anionic and nonionic surfactants,^30^ foam generation temperature and pressure conditions,^28^ the liquid-to-gas ratio within the foam generator,^7,31^ and potentially the use of alternative foam generator designs.^31^

Along with regular foam, the oxidative foam characteristics were also examined to evaluate the influence of SPS oxidant addition on the foam properties in this study. Figure S2 presents the foam stability and quality of the combination between different concentrations of oxidant SPS and surfactants (detailed data are also tabulated in Table S3). The concentrations of AOS, SDS, and TW80 surfactants were fixed at 32, 128, and 17 mM, respectively, with SPS concentration ranging from 10 to 1700 mM. The addition of SPS to the TW80 surfactant solution significantly decreased the foam stability. In contrast, the oxidative foam stability of AOS and SDS surfactants showed only a slight decrease with increasing SPS concentration, except at the highest concentration of 1700 mM. The reduced stability of TW80 foam in the presence of SPS can be attributed to the influence of salt species on nonionic surfactants, which disrupt foam stability.^7,32^ For anionic surfactants such as AOS and SDS, the presence of persulfate (S_2_O_8_^2–^, PS) anions may interact with negatively charged micelles, potentially acting as protective vessels for surfactant anions and preventing their reaction with SPS.^33^ However, the pronounced decrease in foam stability observed at 1700 mM SPS suggests that the oxidative foam stability of all three surfactants was affected by high SPS concentrations. When compared to surfactant solutions without SPS, the oxidative foam stability of AOS, SDS, and TW80 approximately decreased by 2, 5, and 6 times, respectively, in the presence of 1700 mM SPS (see data presented in Table S3). These results indicate that extreme ionic strength limits the feasibility of blending SPS into surfactant solutions for foam generation, particularly for nonionic surfactants.

The results presented in Figure S2 and Table S3 indicate that the oxidative foam quality of the three surfactants remained nearly constant despite increasing SPS concentrations. To validate this observation, images of both regular and oxidative foams were captured using BF and PH microscopes, as shown in Figure S3. Under consistent foam generation conditions, both foam types exhibited uniform overall shapes with minimal differences in foam size. A closer examination of the PH images further revealed that both regular and oxidative foams maintained a consistent size, approximately within the range of 290–400 μm. This uniformity was attributed to the use of a #60 mesh screen (with an opening of 0.25 mm) in the foam generator, ensuring the production of evenly sized foam bubbles.

Furthermore, the thickness of the thin film region in the oxidative foam, formed by the liquid, showed no significant change compared to surfactant foam alone. The measured thin film thickness ranged from 8 to 10 μm for both regular and oxidative foams of anionic surfactants (AOS and SDS) (Figure S3a–d) and from 23 to 27 μm for regular and oxidative foams of the nonionic surfactant (TW80) (Figure S3e,f). These findings suggest that the air and liquid volume percentages in the surfactant foam remained nearly unchanged upon the addition of SPS. Additionally, the foams generated with the nonionic TW80 surfactant exhibited a thicker thin film (Figure S3e,f) due to the lower foam quality of TW80 (96.2%) compared to AOS (99.7%) and SDS foams (98.2%). These results indicate that AOS and oxidative AOS/SPS foams may hold potential for enhancing AS in practical applications. For the second phase of this research, 32 mM AOS, along with 32/50 and 32/1700 mM/mM AOS/SPS, were selected as foaming agents for batch experiments aimed at treating TCE DNAPL in the reactor.

### 3.2 Effect of N_2_ Flow Rate and Mesh Screen of Foam Generator on Foam Characteristics

Figure 4a–c illustrates the impact of the N_2_ flow rate and mesh screen size on the foam properties generated with AOS, SDS, and TW80 surfactants, respectively. The raw data used for these analyses are presented in Table S4. The results indicate that the AOS foam stability increased by approximately 67 min when the N_2_ flow rate was raised from 1.5 to 2.0 L min^–1^. However, further increasing the N_2_ flow rate to 2.5 L min^–1^ led to a significant decrease in the stability by around 130 min. A similar trend was observed for SDS and TW80 foams, as shown in Figure 4b,c, respectively. These findings suggest that an initial increase in the N_2_ input enhances surfactant dynamics at the interface, improving foam stability up to a critical threshold. Beyond this point, as the air flow rate reaches 2.5 L min^–1^, the foam bubbles become increasingly susceptible to rupture, resulting in reduced foam stability. This observation aligns with the study by Varade et al.,^34^ which demonstrated a significant decline in foam stability when the N_2_ flow rate exceeded the optimal level needed to form a critical bubble structure. In light of these results, an air flow rate of 2.0 L min^–1^ was identified as optimal for producing foam with a maximum stability. Therefore, this rate was selected for subsequent experiments examining the influence of foam generator mesh screen size on foam characteristics.

**Figure 4:**
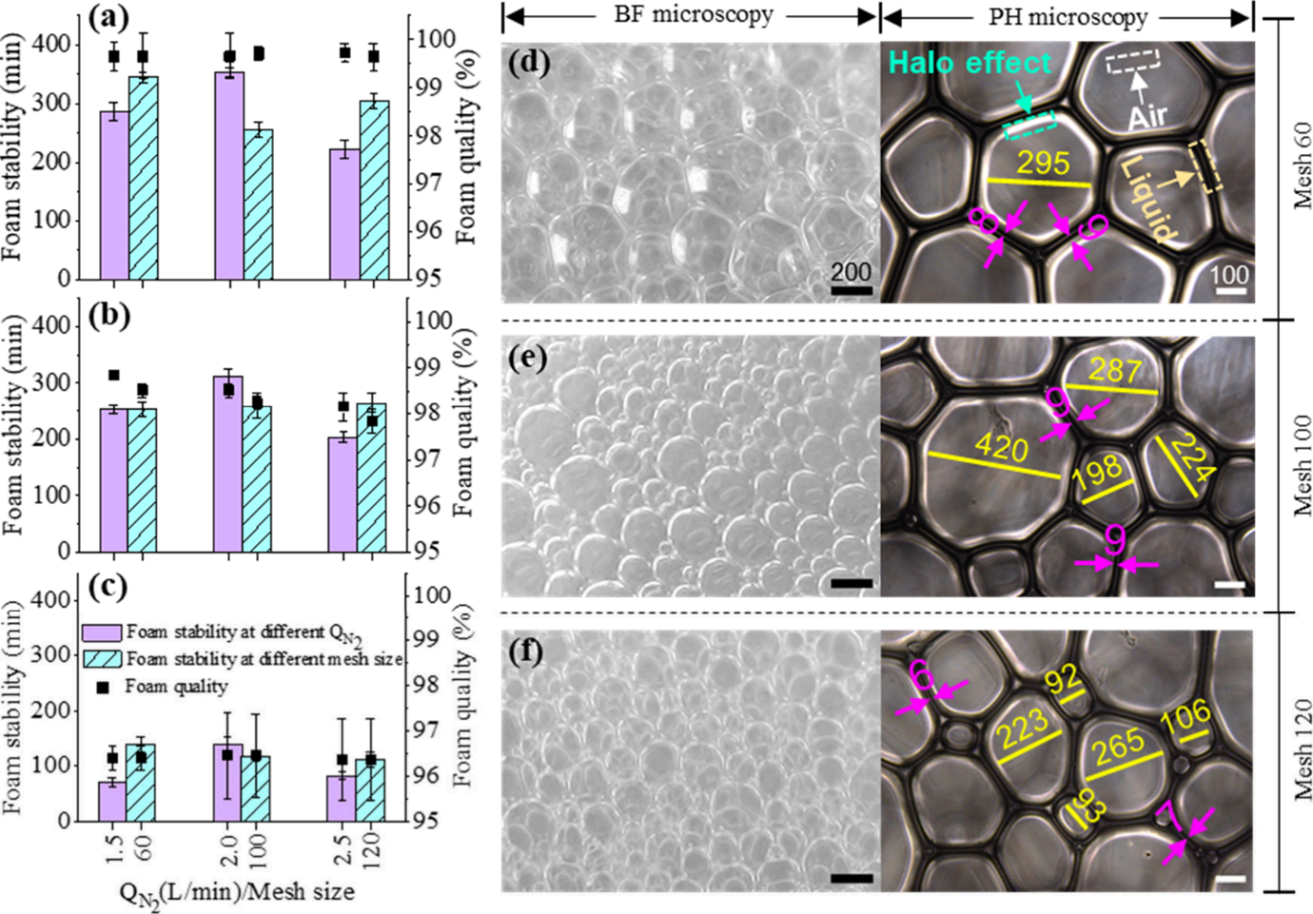
Effect of the N_2_ flow rate and mesh screen size of the foam generator on the properties of foam generated with individual surfactants: (a) AOS, (b) SDS, and (c) TW80. Foam images of AOS were captured using BF and PH microscopy with (d) #60 mesh, (e) #100 mesh, and (f) #120 mesh screens, maintaining a fixed N_2_-to-liquid ratio of 2.0/2.0 (L min^–1^)/L in the foam generator.

Figure 4a–c also reveals that the foam stability generated by all surfactants remained consistent with changes in mesh screen size (no. 60 (0.250 mm), no. 100 (0.149 mm), and no. 120 (0.125 mm)). Similarly, the foam quality of all surfactants exhibited minimal variation (±0.2–1.0%) despite variations in N_2_ flow rate or mesh screen size. This observation is further supported by measurements of the thin film thickness of foam bubbles, which remained stable in the range of 6–9 μm, as shown in Figure 4d–f.

In general, the size of the AOS foam decreased noticeably with a reduction in mesh screen size. Bright-field microscopy images revealed that most of the foam exhibited a uniform size after passing through the mesh screen. As shown in Figure 4, when using the #60 mesh screen, AOS foam bubbles typically ranged from approximately 290 to 400 μm (Figures 4d and S3). In contrast, small foam bubbles were observed with the #100 and #120 mesh screens, with sizes ranging from approximately 195 to 225 μm and 90 to 110 μm, respectively (see Figure 4e,f). However, large foam bubbles (∼420 and ∼265 μm, respectively) were occasionally seen with the #100 and #120 mesh screens, likely due to contact with surrounding air as the foam exited the generator. These findings suggest that the mesh screen size of the foam generator significantly influences both the foam size and the characteristics. Based on these results, an N_2_ flow rate of 2.0 L min^–1^ and a #60 mesh screen were selected for the treatment of TCE DNAPL in the aqueous phase.

### 3.3 FEAS for Treatment of TCE DNAPL in Aqueous Phase

The relationship between the flow rate injected into the FEAS reactor and the outflow rate for discharge, along with the observed reactor pressure, is shown in Figure S4. The calculated total flow rates for injection and outflow closely match the N_2_ flow rate generated by the foam generator (∼2.0 L min^–1^). Reactor pressure increases from approximately 30 mmAq (0.04 psi) to 135 mmAq (0.19 psi) as the injection flow rate rises from 500 to 1900 mL min^–1^, demonstrating that a higher N_2_ injection leads to increased reactor pressure. The relationship between reactor pressure and injection flow rate is described by a linear equation (inset of Figure S4, *R* ^2^ = 0.99). The top of the reactor was sealed with a cover, incorporating a GAC adsorption tube open at both ends, to maintain internal pressure below 30 mmAq. Consequently, N_2_ or foam flow rates of 100, 200, and 300 mL min^–1^ were selected for the safe treatment of TCE DNAPL in the aqueous phase, as outlined in Table S5.

To assess the performance of FEAS in TCE dissolution, volatilization, and degradation under varying conditions, a series of experiments was conducted in a batch reactor. Figure 5 illustrates TCE mass distributions under different N_2_/foam flow rates across various experimental setups, including N_2_ injection alone, solutions containing either AOS or SPS/AOS surfactants, and injections with both regular and oxidative foams. As expected, no TCE degradation was observed during the 2 h operation in any of the control tests, including N_2_ sparging (Figure 5a-1–a-3), AOS surfactant (Figure 5b-1–b-3), or surfactant/SPS (Figure 5c-1–c-3). The percentages of total TCE mass in the dissolved and gas phases ranged from 4.68 to 8.17%, 5.22 to 11.29%, and 5.50 to 9.44% for experimental groups a–c, respectively, as shown in Figure 5. Approximately 80% to 90% of the TCE mass remained as DNAPL in the aqueous solution, with less than 10% TCE loss observed in the mass balance analysis. Additionally, Figure 6 presents the temporal changes of TCE mass in the aqueous phase (dissolved phase) and gas phase (headspace). The limited TCE solubilization in water over a short period likely hindered any potential activities such as volatilization or oxidation within the aqueous phase.^35^

**Figure 5:**
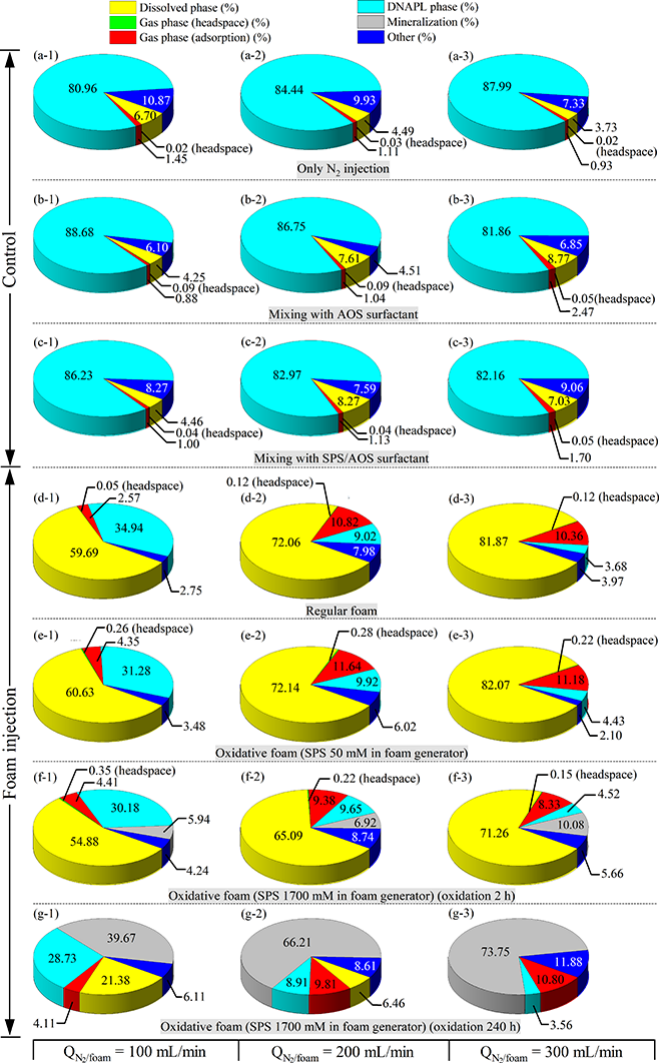
Mass distribution of TCE concentrations on different control and foam injection tests for 2 h of operation ((a-1) to (f-3)) and 240 h of operation ((g-1) to (g-3)). Experimental conditions are summarized in Table S5.

**Figure 6:**
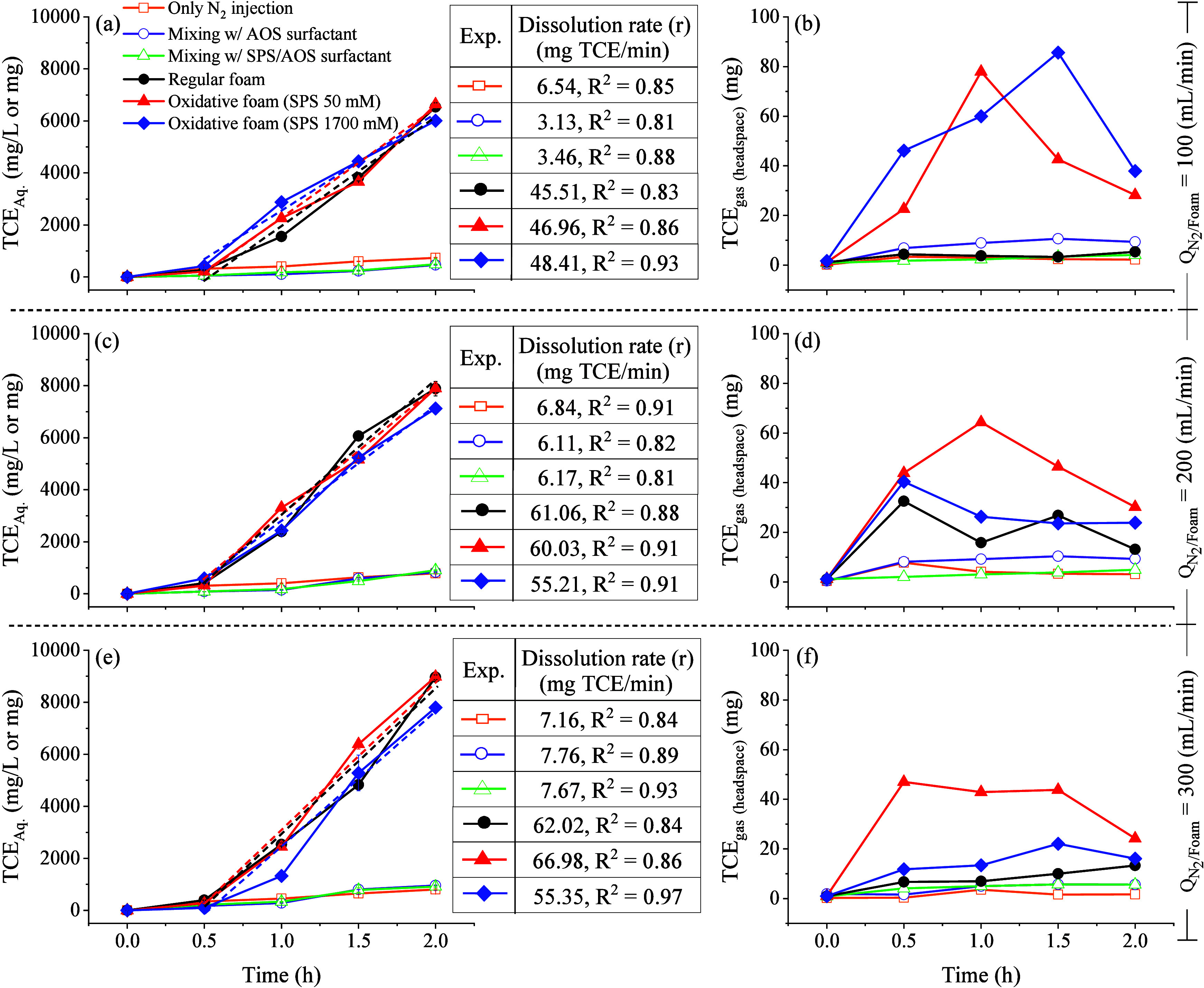
TCE mass in the aqueous phase and gas phase (headspace) under foam flow rate of (a, b) 100 (mL min^–1^), (c, d) 200 (mL min^–1^), and (e, f) 300 (mL min^–1^) for 2 h of operation. The reactor volume is 1.0 L.

The results in Figure 5d-1–d-3 showed that approximately 60–82% of TCE DNAPL was dissolved upon injection of foam and the extent of TCE dissolution increased with increased foam injection flow rate, as compared to 4–7% observed with injection of N_2_ alone after 2 h of injection (Figure 5a-1–a-3), and it was even higher than those with the initial presence of AOS surfactant or SPS/AOS in aqueous solution throughout 2 h mixing (Figure 5b-1–b-3,c-1–c-3). AOS concentration in the reactor was calculated as approximately 1.5, 3.1, and 4.6 mM with foam injection for 2 h at 100, 200, and 300 (mL min^–1^), respectively (Table S6). When the surfactant concentration in water exceeds several times the CMC, surfactant molecules predominantly adsorb at interfaces as monomers. The enhanced dissolution of TCE through surfactant foam injection, compared with conventional surfactant dissolution in water, can be attributed to the formation of a thin surfactant-containing foam layer at the gas–liquid interface, which enhances interfacial interactions, enabling TCE molecules to adsorb onto foam surfaces. This adsorption increases the extent of TCE partitioning into foam bubbles, where the surfactant-stabilized interface provides a favorable environment for TCE volatilization into the gas phase within the bubbles. In the lamella region of the foam, TCE remains associated with the surfactant layer, leading to increased dissolution and volatilization. This mechanism explains the observed enhancement of TCE DNAPL removal in the FEAS process (see in Figure S5).

Oxidative foam injection with 50 mM SPS resulted in a TCE mass distribution (Figure 5e-1–e-3) similar to that observed in AOS foam experiments (Figure 5d-1–d-3). TCE concentrations in the aqueous and gas phases also mirrored those in AOS foam tests, with consistent dissolution patterns as the foam flow rate increased (Figure 5d). The dissolution rate (*r*) of TCE mass in the dissolved phase for both regular and oxidative foams was determined from the linear trend line and shown in Figure 6 for three foam injection rates. At a foam injection rate of 100 mL min^–1^ (Figure 6a), the TCE dissolution rate remained similar for both regular and oxidative foam injection, ranging from 46 to 48 mg of TCE/min. However, as the foam injection rate increased, the dissolution rate correspondingly rose to 55–67 mg TCE/min at 300 mL min^–1^. These results suggest that the low SPS concentration had a minimal impact on foam properties, allowing oxidative foam to effectively dissolve TCE DNAPL under identical operational conditions. However, the SPS concentration may have been insufficient to significantly enhance TCE degradation, likely due to the limited reaction time within the reactor. To address this, additional experiments were conducted using oxidative foam injection with a higher SPS concentration in the foam generator (1700 mM) after 2 h of injection, extending the oxidant retention time in the reactor to 240 h. This adjustment aligns with the recommended reaction time of approximately 200–400 h for near-complete SPS oxidation of TCE (SPS/TCE molar ratio of 50:1 at an initial TCE concentration of 0.46 mM) in the aqueous phase under ambient temperatures of 20–30 °C.^36^

The TCE mineralization mass during a 2 h operation ranged from approximately 6% to 10% using oxidative foam (1700 mM SPS) at various flow rates, while TCE dissolution reached 55–71%, as shown in Figure 5f-1–f-3. Mineralization in this study refers to the complete breakdown of the TCE molecule into inorganic end products, specifically, chloride ions (Cl^–^). Accordingly, mineralization was assessed using the Cl^–^/TCE molar ratio (3:1), which reflects the release of three mol of chloride ions per mole of TCE degraded in the aqueous phase. It should be noted that GC/MS analysis of postreaction samples (at 2 and 240 h) was conducted using NIEA Method W785-54 B, which targets 60 standard VOCs, including dichloroethylene and vinyl chloride. The results showed that, aside from the parent compound TCE, no other compounds were detected above the quantitation detection limit (0.0010 mg/L). Accordingly, the mass balance reflects only the disappearance of TCE, as no transformation products were observed within the detection limits of the method. Once TCE DNAPL dissolved into the aqueous phase, PS or SO_4_^•–^ generated from PS (eq 3) facilitated TCE mineralization via eqs 4 and 5.

$$S_{2}{O_{8}}^{2-}\overset{\begin{array}{c} Thermal\;Activation\\ Ambient\;∼99^{{}^{\circ}}C \end{array}}{\to }2{{\mathrm{SO}}_{4}}^{•-}$$ 3

$$6{{\mathrm{SO}}_{4}}^{•-}+C_{2}{\mathrm{HCl}}_{3}+4H_{2}O\to 6{{\mathrm{SO}}_{4}}^{2-}+2{\mathrm{CO}}_{2}+9H^{+}+3{\mathrm{Cl}}^{-}$$ 4

$$3S_{2}{O_{8}}^{2-}+C_{2}{\mathrm{HCl}}_{3}+4H_{2}O\to 6{{\mathrm{SO}}_{4}}^{2-}+2{\mathrm{CO}}_{2}+9H^{+}+3{\mathrm{Cl}}^{-}$$ 5

Notably, TCE mineralization increased to 40–74% when using oxidative foam with a higher SPS concentration (1700 mM in the foam generator) and an extended reaction time of 240 h, as illustrated in Figure 5g-1–g-3. The observed TCE degradation kinetics are attributed to sulfate radicals generated via the intrinsic thermal activation of SPS at ambient temperature (25 ± 2 °C), often referred to as self-activation. This process involves homolytic cleavage of the peroxide bond in the persulfate ion, producing two sulfate radicals. While this reaction proceeds more rapidly at higher temperatures, it still occurs slowly at ambient temperature, accounting for the degradation kinetics observed and modeled in this study. Liang and Bruell^21^ reported a TCE degradation rate constant (*k*_1_) of 5.59 × 10^–4^ mM^0.2^ min^–1^ by SPS at 40 °C. Using the Arrhenius equation (eq 6), this value was adjusted to 25 °C, yielding a degradation rate of 6.90 × 10^–5^ (mM^0.2^ min^–1^). The TCE degradation kinetics under these conditions can be described by eq 7.

$$\ln\frac{k_{2}}{k_{1}}=-\frac{E_{a}}{R}\left(\frac{1}{T_{2}}-\frac{1}{T_{1}}\right)$$ 6

where *E*_a_ (J mol^–1^) is activation energy (*E*_a_ = 108130 J mol^–1^), *R* (J mol^–1^ K^–1^) is ideal gas constant (*R* = 8.314 J mol^–1^ K^–1^),^21^ *T*_1_ (K) is temperature at 40 °C (*T*_1_ = 313 K), and *T*_2_ (K) is temperature in the reactor (*T*_2_ = 298 K).

$$-d\left[\mathrm{TCE}\right]/dt=\left(6.90\times 10^{-5}{\mathrm{mM}}^{0.2}\min^{-1}\right){\left[\mathrm{TCE}\right]}^{0}{\left[S_{2}{O_{8}}^{2-}\right]}^{0.8}\left(@25^{{}^{\circ}}C\right)$$ 7

Upon injection of oxidative foam containing 1700 mM SPS from a foam generator for 2 h at different flow rates, the aqueous SPS concentration reached 82–245 mM, as presented in Table S6. Using eq 6 with *k*_1_ (at 25 °C), the TCE degradation rate (−d­[TCE]/d*t*) was calculated as 5.62 × 10^–3^ mM min^–1^ in the presence of 245 mM SPS at the end of the foam injection (300 mL min^–1^ for 2 h), as shown in Table S7 . Figure S6 illustrates the accumulated SPS concentration over time to support these data. A comparison of predicted and experimental mineralization percentages indicates that, at lower foam injection rates (100 and 200 mL min^–1^), the predicted mineralization closely matched the experimental values. However, at 300 mL min^–1^, the predicted mineralization (97%) exceeded the experimentally observed value (74%), suggesting possible interferences, likely due to the higher injected surfactant concentration reducing the extent of TCE degradation. After an additional 240-h reaction period (Figure S6), the SPS concentrations decreased to 1.8, 11.4, and 71.2 mM for flow rates of 100, 200, and 300 mL min^–1^, respectively, aligning with the predicted values from eq 7. These results confirm that chemical oxidation between SPS and TCE occurred within an extended retention time. Moreover, the oxidative foam not only enhances the dissolution and volatilization of TCE DNAPL but also facilitates TCE degradation through oxidation. This demonstrates that FEAS has significant potential to improve conventional AS techniques. Kim et al.^37^ investigated pulsed air sparging (PAS) as a remediation technology for TCE-contaminated groundwater in a sandy aquifer through laboratory experiments. Their results demonstrated that PAS effectively removed most of the dissolved TCE and predicted up to 95% removal of residual TCE mass after operation for 24 h of operation. However, PAS primarily achieves removal through phase transfer mechanisms, dissolution, and volatilization, rather than destruction.

In contrast, the FEAS system developed in this study achieved substantial mineralization of TCE (approximately 74% at 1700 mM SPS), indicating true contaminant degradation rather than simple phase transfer. The high efficiencies of dissolution (55–82%) and volatilization (3–12%) observed within just 2 h using the oxidative AOS/SPS foam highlight its ability to enhance mass transfer, aligning with the goals of PAS. However, the key advantage of the FEAS/SPS system lies in its capacity for in situ chemical destruction and mineralization of TCE, offering a more comprehensive remediation approach that combines mobilization, dissolution, and degradation.

Additionally, Yan and Schwartz^38^ studied the kinetics and mechanisms of TCE oxidation by permanganate in aqueous solution. Their findings indicated that the reaction typically follows second- or pseudo-first-order kinetics with respect to both TCE and permanganate concentrations. This behavior differs from the kinetics observed in the current FEAS/SPS system treating TCE DNAPL, where degradation followed zero-order kinetics with respect to TCE and 0.8 order with respect to SPS concentration (eq 7).

The observed zero-order dependence on TCE suggests that, under these experimental conditions, where a DNAPL source is present and foam-enhanced delivery is applied, the overall reaction rate was limited by the dissolution of TCE from the DNAPL phase into the aqueous phase rather than the intrinsic chemical reaction rate with activated SPS. In contrast, the higher-order kinetics observed by Yan and Schwartz^38^ reflect systems where TCE is already dissolved and homogeneously available, making the reaction rate the limiting factor.

While both oxidants can achieve high levels of mineralization, the kinetic insights from this study emphasize the importance of enhancing DNAPL dissolution to accelerate degradation. This underscores the strength of the FEAS system in addressing rate limitations imposed by the DNAPL phase and highlights its potential for more effective in situ chemical oxidation of dense-phase contaminants.

Additionally, the ORP of approximately 450–700 mV was recorded in the solution of SPS/AOS experiments (Figure 7a-3,b-2,b-3), implying higher oxidizing conditions as compared to approximately 210–250 mV in the other experiments without adding SPS (Figure 7a-1,a-2,b-1). In the experiments with SPS, solution pH values slightly decreased to ∼5.3–6.7 (Figure 7a-3,b-2,b-3), but pH level further decreased to ∼3.2 after the extended reaction of 240 h. These results stem from the inherent behavior of SPS upon contact with water, where it releases hydrogen sulfate (HSO_4_^–^), which subsequently dissociates to produce H^+^.^18^ Moreover, SO_4_^•–^, generated during SPS activation can further react with water, producing both H^+^ and hydroxyl radical (^•^OH), thereby contributing to a decrease in pH. The corresponding reaction equations are provided in Table S8. Note that DO values were stably present within the range of 6–9 mg/L in the system for all experiments.

**Figure 7:**
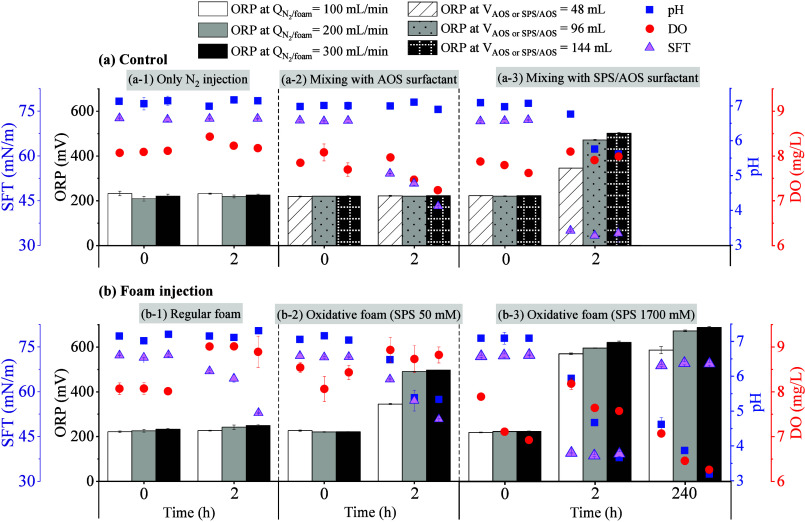
Variation of pH, ORP, DO, and SFT on the different control and foam injection tests.

The SFT of the solution was measured and is illustrated in Figure 7. The SFT decreases from ∼72.2 ± 0.8 mN/m in water to ∼45.1 ± 12.0 mN/m in the AOS addition or foam injection experiments, which are similar to the SFT of approximately 30–40 mN/m of AOS surfactant reported in the AOS concentration range of 1–10 mM at 25 °C by Farajzadeh et al.^39^ The lower SFT contributes to increasing the solubility of nonpolar molecules in the aqueous phase and also may enhance the stability of foam, dispersions, and emulsions. However, the SFT of solution in the oxidative foam experiment increased to ∼69.5 mN/m after 240 h (Figure 7b-3). This is possibly due to the presence of such a high concentration of SPS salt, which oxidized the surfactant and reduced its activity.^40^

## 4 Conclusions

This study investigates the characteristics of various foams generated by using individual surfactants (TW80, SDS, and AOS) and surfactant combinations (TW80/SDS and TW80/AOS). The foams were evaluated to identify the most suitable surfactant for FEAS in treating TCE DNAPL in the aqueous phase. Foams with and without the SPS oxidant were analyzed for their effectiveness. The results indicated that AOS foam exhibited the highest stability and quality (345 min and 99.7% at 32 mM), outperforming SDS (240 min and 98.5% at 128 mM), TW80 (150 min and 96.9% at 16.8 mM), TW80/AOS (10 min and 94.8% at 16.8 mM/32 mM), and TW80/SDS (6 min and 94.3% at 16.8 mM/128 mM). Under experimental conditions using a foam generator with a #60 mesh screen and an N_2_/liquid ratio of 2.0/2.0 (L min^–1^)/L, an idealized AOS foam exhibited a bubble size range of 290–400 μm and a thin film thickness of 6–9 μm. Based on these findings, regular AOS foam (32 mM) and oxidative foam (AOS with 50 or 1700 mM SPS oxidant) were used in FEAS experiments within a laboratory-scale reactor. After 2 h of operation at various foam flow rates, TCE dissolution and volatilization reached 55–82% and 3–12%, respectively, when using AOS foam (32 mM) or oxidative foam (AOS/SPS at 32/50 or 32/1700 mM/mM). In contrast, control tests, including N_2_ injection alone, AOS (32 mM) surfactant solution, or AOS/SPS (32 mM/50 mM) solution, achieved only 4–9% dissolution and 1–3% volatilization. Furthermore, TCE mineralization reached ∼74% in the presence of a high SPS oxidant concentration (1700 mM), with a degradation rate of 5.62 × 10^–3^ mM min^–1^ at a foam injection flow rate of 300 mL min^–1^ after 240 h of oxidation. The kinetic rate equation was determined as −d­[TCE]/d*t* = (6.90 × 10^–5^ mM^0.2^ min^–1^) [TCE]^0^[S_2_O_8_^2–^]^0.8^ at 25 °C. Overall, this study demonstrates that FEAS is a promising technique for enhancing conventional AS by improving TCE DNAPL removal through dissolution, volatilization, and oxidation processes in aqueous systems. In practical field applications, the FEAS system would involve injecting optimized oxidative foam into the contaminated groundwater zone through remediation wells. A critical aspect of implementation relates to both the observed volatilization of TCE within the foam and its subsequent degradation by the oxidant. As the foam rises through the saturated zone, volatile TCE can partition into the gas phase within the foam bubbles. Upon reaching the vadose zone, the vapor-phase TCE may migrate upward. Therefore, in full-scale or pilot field deployments of FEAS for TCE DNAPL remediation, it is highly recommended and, in many cases, necessary to pair FEAS injection in the saturated zone with a SVE system in the overlying vadose zone. This integrated FEAS-SVE approach provides comprehensive control over the total mass of TCE removed from the DNAPL source, preventing its re-entry into the groundwater or release into the atmosphere. Moreover, it enables effective accounting for both the portion degraded via oxidative processes and the portion removed via volatilization, ensuring safe and complete contaminant management.

## Supplementary Material



## Data Availability

Data will be made available on request.

## Notes

Flowchart of the experimental design matrix (Figure S1); Characteristics of oxidative foams generated with different surfactants (Figure S2); Foam images of different foams captured using bright-field (BF) and phase-contrast (PH) microscopes (Figure S3); The variation of reactor pressure (*P*_reactor_) with the relationship between reactor injected flow rate (*Q*_N2_) and out flow rate in the reactor (Figure S4); The mechanism of surfactant foams might enhance TCE dissolution and volatilization (Figure S5); The variation of SPS concentration on different control and foam injection tests (Figure S6); Physicochemical properties of surfactants used in this study (Table S1); Characteristics of regular foams generated with different surfactants concentrations (Table S2); Characteristics of oxidative foams generated with different surfactants (Table S3); Effects of N_2_ flow rate and mesh size on foam properties (Table S4); Experimental design for batch experiments in the reactor for remediating TCE DNAPL contamination (Table S5); Concentration of AOS and SPS with foam injection for 2 h at different foam flow rates (Table S6); Comparison of prediction and oxidative foam injection experiment with 1700 mM SPS after 240 h for TCE mineralization (Table S7); Reactions involved in the degradation of SPS in aqueous media (Table S8) (PDF)
