# Supplementary material for: Comparative Assessment of Regular and Persulfate Oxidative Foams in Air Sparging for Trichloroethylene Dense Nonaqueous Phase Liquid Remediation
Source: ACS Environ Au. 2025 Jun 25;5(5):468–78. doi: 10.1021/acsenvironau.5c00046 (PMC12447226; doi:10.1021/acsenvironau.5c00046)
Supplement: Supplementary file 1 [file vg5c00046_si_001.pdf]

Supporting Information

Comparative Assessment of Regular and Persulfate Oxidative Foams in Air Sparging for Trichloroethylene Dense Nonaqueous Phase Liquid Remediation

Xuyen Thi Hong Luong, Chenju Liang*

Department of Environmental Engineering, National Chung Hsing University

145 Xingda Road, Taichung 402, Taiwan

*Corresponding author. Tel.: +886-4-22856610; Fax: +886-4-22856610

Chenju Liang email: cliang@nchu.edu.tw

Xuyen Thi Hong Luong email: xuyenluong@yahoo.com

Contents

Figure S1. Flowchart of the experimental design matrix.

Figure S2. Characteristics of oxidative foams generated with different AOS/SPS, SDS/SPS, and TW80/SPS concentration ratios.

Figure S3. Foam images of (a) AOS, (b) AOS/SPS, (c) SDS, (d) SDS/SPS, (e) TW80, and (f) TW80/SPS were captured using bright-field (BF) and phase-contrast (PH) microscopes under the experimental conditions of foam generator were fixed at a #60 mesh screen and an N_2/liquid ratio of 2.0/2.0 (L min^{-1})/L. Black scale bar represents 200 µm and white scale bar represents 100 µm. Note: the concentrations of AOS, SDS, and TW80 are 32, 128, 16.8 mM, respectively.

Figure S4. The variation of reactor pressure ($P_{reactor}$)with relationship between reactor injected flow rate (Q_{N2}) and out flow rate in the reactor.

Figure S5. The mechanism of surfactant foams might enhance TCE dissolution and volatilization in this study.

Figure S6. The variation of SPS concentration on different control and foam injection tests.

Table S1. Physicochemical properties of surfactants used in this study.

Table S2. Characteristics of regular foams generated with different surfactants concentrations.

Table S3. Characteristics of oxidative foams generated with different AOS/SPS, SDS/SPS, TW80/SPS concentrations ratios.

Table S4. Effects of N_2 flow rate and mesh size on foam properties.

Table S5. Experimental design for batch experiment in the reactor for remediating TCE DNAPL contamination.

Table S6. Concentration of AOS and SPS with foam injection for 2 h at different foam flow rate.

Table S7. Comparison of prediction and oxidative foam injection experiment with 1700 mM of SPS after 240 h for TCE mineralization.

Table S8. Reactions involved in the degradation of SPS in aqueous media.

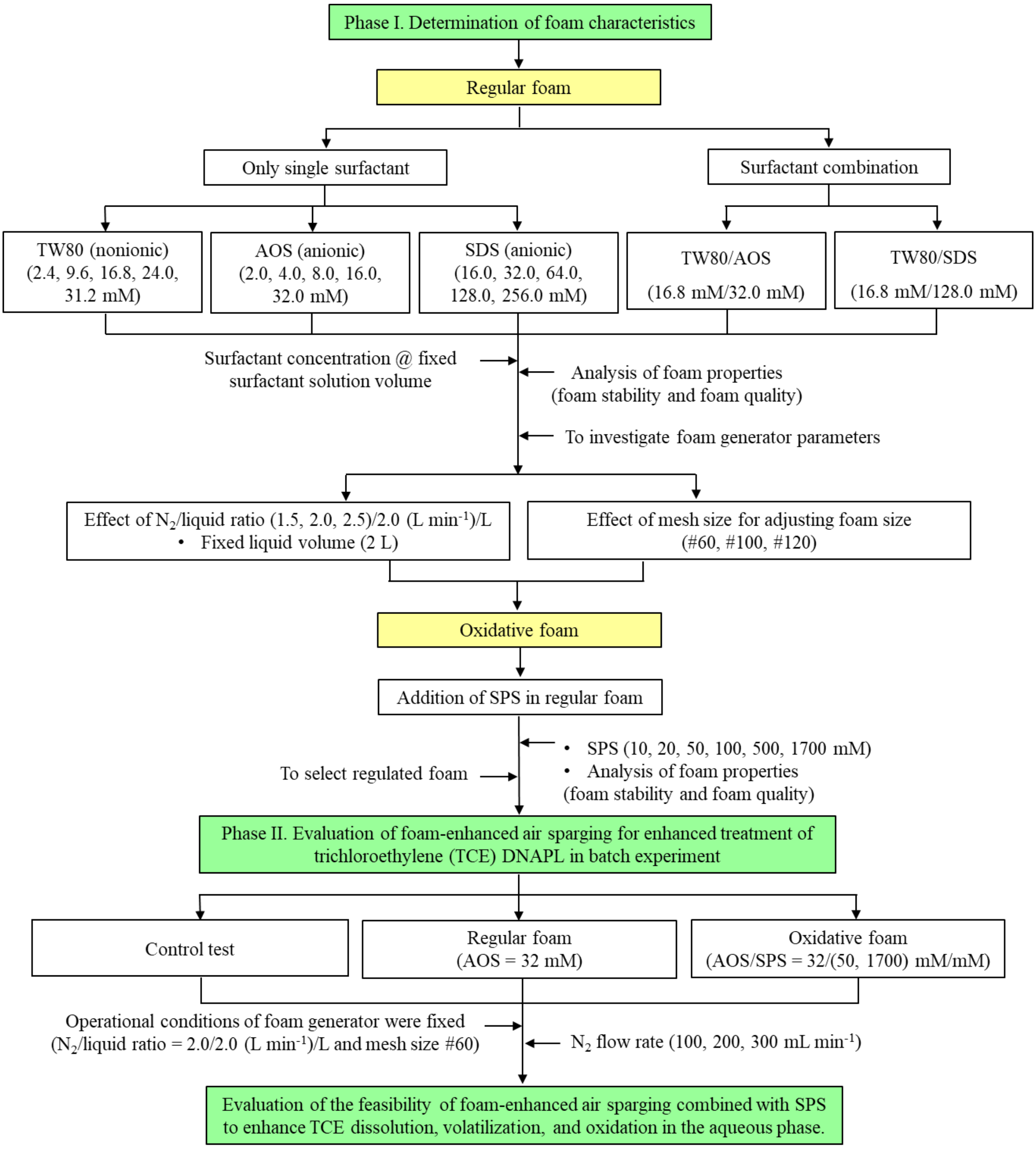

Figure S1. Flowchart of the experimental design matrix.

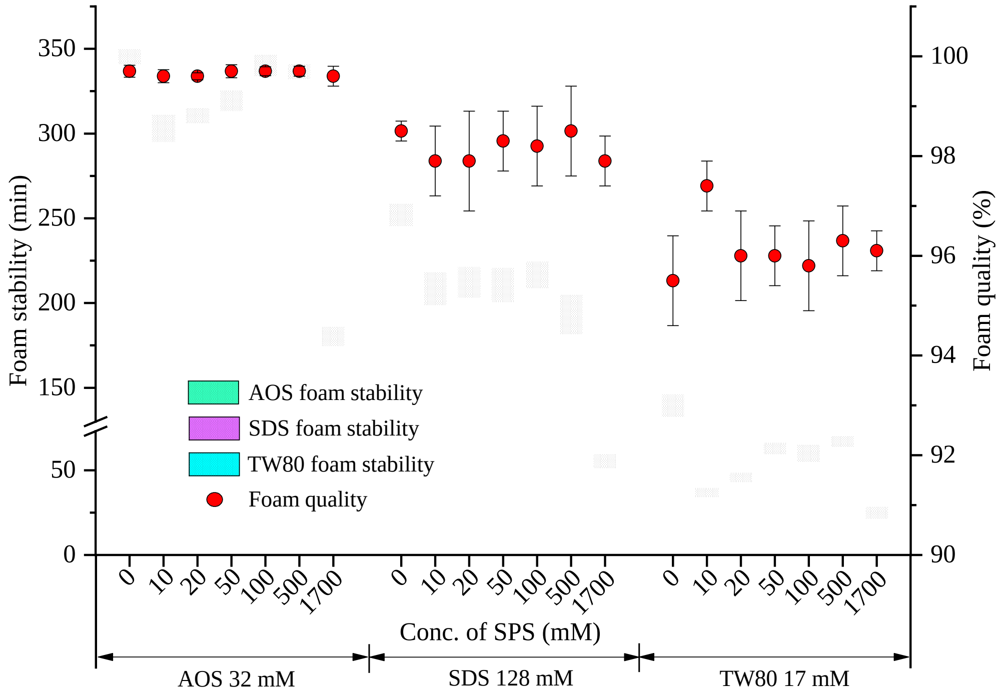

Figure S2. Characteristics of oxidative foams generated with different AOS/SPS, SDS/SPS, and TW80/SPS concentration ratios.

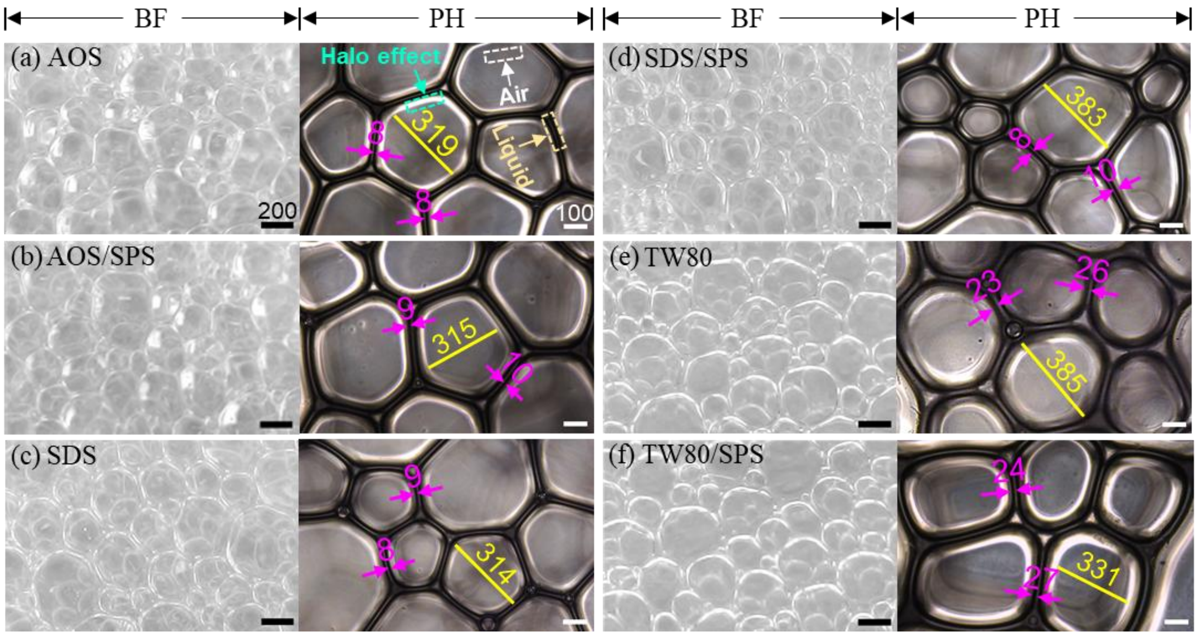

Figure S3. Foam images of (a) AOS, (b) AOS/SPS, (c) SDS, (d) SDS/SPS, (e) TW80, and (f) TW80/SPS were captured using bright-field (BF) and phase-contrast (PH) microscopes under the experimental conditions of foam generator were fixed at a #60 mesh screen and an N_2/liquid ratio of 2.0/2.0 (L min^{-1})/L. Black scale bar represents 200 μm and white scale bar represents 100 μm. Note: the concentrations of AOS, SDS, and TW80 are 32, 128, 16.8 mM, respectively.

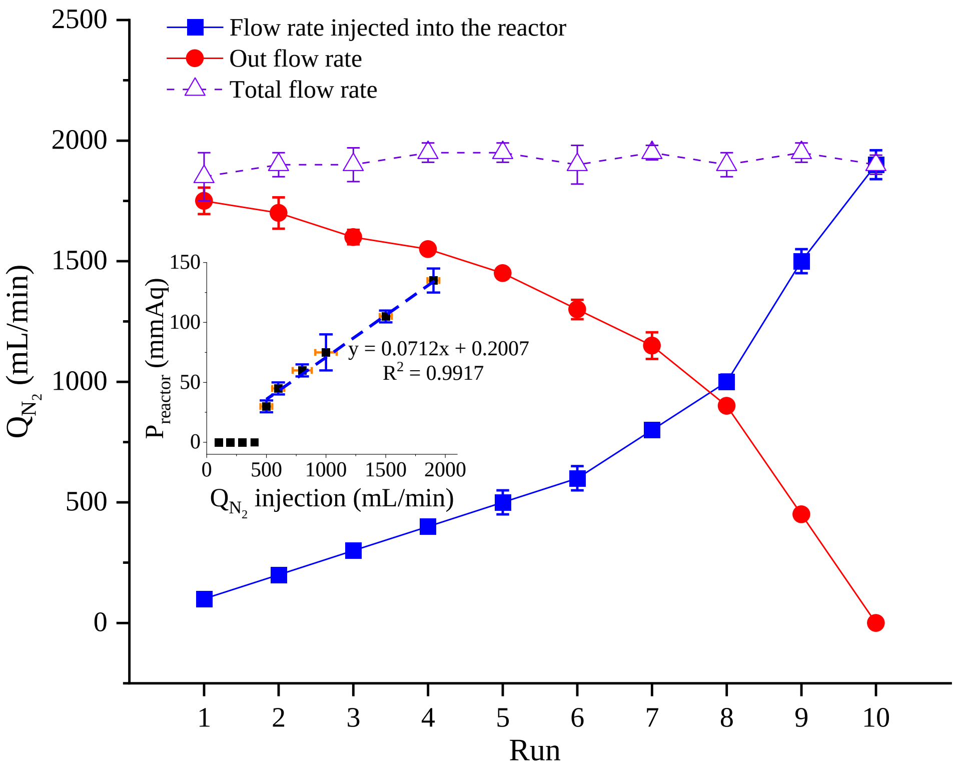

Figure S4. The variation of reactor pressure ($P_{reactor}$) with relationship between injected flow rate (Q_{N2}) and out flow rate in the reactor.

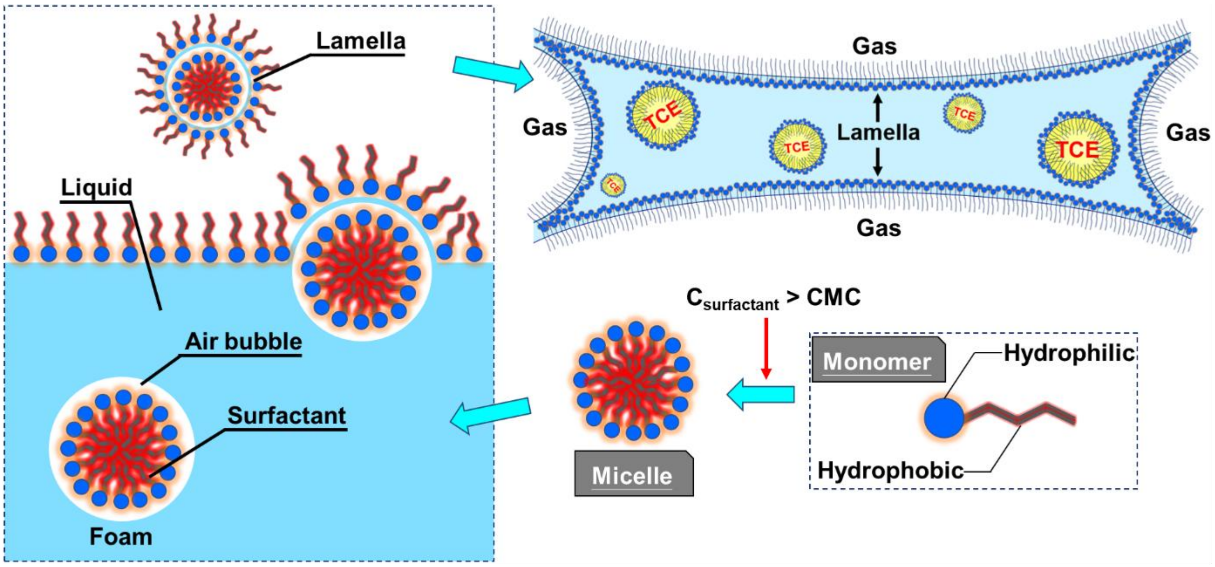

Figure S5. The mechanism of surfactant foams might enhance TCE dissolution and volatilization in this study.

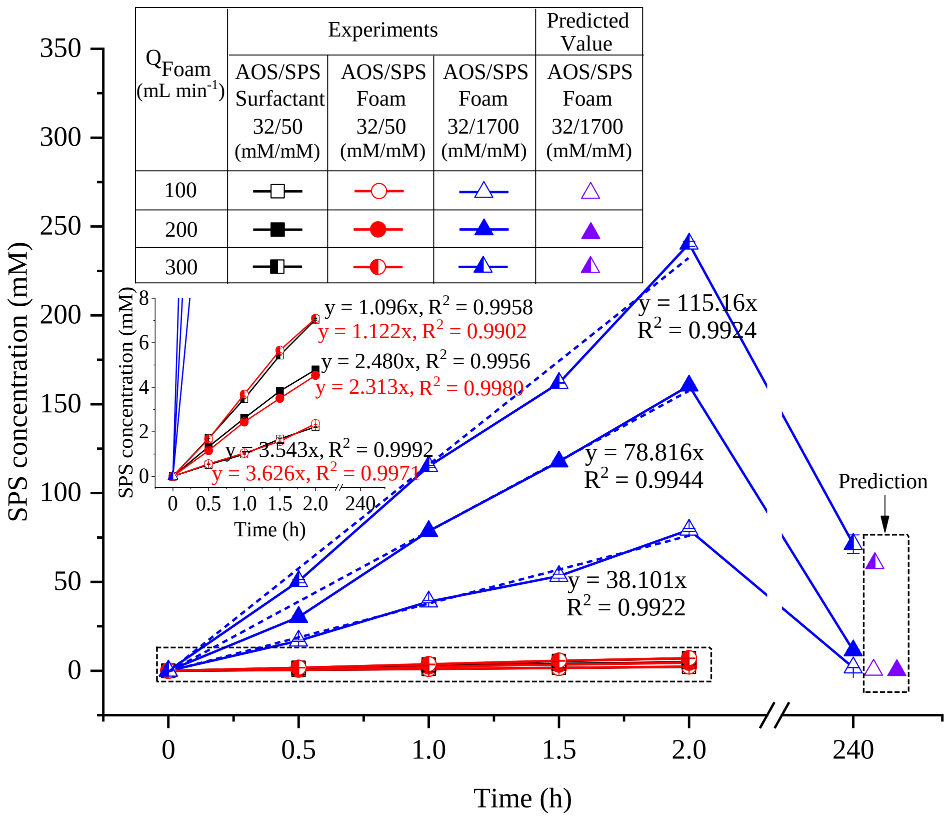

Figure S6. The variation of SPS concentration on different control and foam injection tests.

Table S1. Physicochemical properties of surfactants used in this study.

Properties	Surfactants used in this study		
	Polyoxyethylene (20) sorbitan monooleate TW80 [1,2]	Sodium dodecyl sulfate SDS [3]	Sodium α-olefin sulfonate AOS [4]
Ionic type	Nonionic	Anionic	Anionic
CAS No.	9005-65-6	151-21-3	68439-57-6
Density (g/mL) @25°C	1.06	1.01	1.07
M.W. (1) (g/mol)	1,310	288.372	298.417
CMC (2) (mM)	0.012	8.1	1.0
Solubility in water (mg/mL)	106	200	Miscible
HLB (3)	15.0	40	11–16
Cloud point (^{o}C)	65	>100	7
Aggregation number	60	62	76–128

Noted: (1): molecular weight of surfactant; (2): critical micelle concentration; and (3): hydrophilic lipophilic balance of surfactant

Table S2. Characteristics of regular foams generated with different surfactants concentrations.

Type of surfactant	Surfactant	CMC (mM)	Times × CMC	Experimental concentration (mM)	FS (min)	SD (min)	FQ (%)	SD (%)	CA (°)	SD (°)
Anionic	Sodium α-olefin sulfonate (AOS)	1.0 [4]	2	2.0	222.2	5.0	99.6	0.1	20.9	4.0
			4	4.0	248.1	9.7	99.6	0.2	15.8	1.9
			8	8.0	303.0	8.7	99.7	0.1	13.3	3.8
			16	16.0	343.6	7.1	99.6	0.1	11.9	3.4
			32	32.0	345.4	8.3	99.7	0.1	12.6	2.9
	Sodium docecyl sulfate (SDS)	8.1 [3]	2	16.0	197.2	9.7	97.6	0.1	16.6	3.2
			4	32.0	241.2	7.4	98.6	0.2	15.3	3.5
			8	64.0	247.1	8.3	98.5	0.3	14.4	3.2
			16	128.0	252.0	12.3	98.5	0.1	11.1	3.5
			32	256.0	251.0	10.7	98.6	0.2	11.6	2.2
Nonionic	Polyoxythyl -ene (20) sorbitan mono (TW80)	0.012 [2]	200	2.4	23.9	5.5	93.9	2.2	26.8	3.1
			800	9.6	140.6	9.2	96.9	0.9	19.2	3.6
			1400	16.8	139.4	12.9	96.5	0.9	15.3	3.8
			2000	24.0	138.9	14.9	95.7	0.9	12.4	2.1
			2600	31.2	147.2	7.9	97.0	0.6	13.5	1.2
Nonionic/ Anionic	TW80/AOS		1400/32	16.8/32.0	10.0	1.1	94.8	0.2	17.4	2.4
	TW80/SDS		1400/16	16.8/128.0	6.1	2.0	94.3	0.2	17.1	3.4

Note: the experimental conditions of foam generator were fixed at screen #60 mesh and N_2/liquid ratio is 2.0/2.0 (L min^{-1})/L). FS represents foam stability (min), FQ represents foam quality (%), CA represents contact angle of surfactant solution on quartz glass (degrees), and SD represents standard deviation (n = 3).

Table S3. Characteristics of oxidative foams generated with different AOS/SPS, SDS/SPS, TW80/SPS concentrations ratios.

Type of surfactant	Surfactant/SPS	[Surfactant] (mM)	[SPS] (mM)	FS (min)	SD (min)	FQ (%)	SD (%)
Anionic	AOS/SPS	32.0	0	345.2	8.3	99.7	0.1
			10	303.1	15.8	99.6	0.2
			20	310.2	8.7	99.6	0.1
			50	319.3	11.7	99.7	0.1
			100	340.1	12.3	99.7	0.1
			500	336.6	8.6	99.7	0.1
			1700	180.1	10.9	99.6	0.2
	SDS/SPS	128.0	0	252.0	13.3	98.5	0.2
			10	208.3	19.2	97.9	0.7
			20	212.2	17.9	97.9	1.0
			50	210.7	20.0	98.3	0.6
			100	216.5	15.3	98.2	0.8
			500	193.0	23.1	98.5	0.9
			1700	55.2	8.5	97.9	0.5
Nonionic	TW80/SPS	16.8	0	139.4	12.9	95.5	0.9
			10	36.7	5.0	97.4	0.9
			20	45.6	5.3	96.0	0.5
			50	62.6	6.7	96.0	0.9
			100	60.0	10.0	95.8	0.6
			500	66.7	5.8	96.3	0.7
			1700	25.0	6.7	96.1	0.4

Note: the experimental conditions of foam generator were fixed at screen #60 mesh and air/liquid ratio is 2.0/2.0 (L min^{-1})/L. FS represents foam stability (min), FQ represents foam quality (%), and SD represents standard deviation (n = 3).

Table S4. Effects of N_2 flow rate and mesh size on foam properties.

Parameters			Experimental objectives					
			Effect of N_2 flow rate			Effect of mesh screen size		
N_2 flow rate (L min^{-1})			1.5	2.0	2.5	2.0		
Mesh screen size			60			60	100	120
Foam characteristics	AOS	FS (min)	286.7	354.0	223.3	345.2	255.2	305
		SD (min)	15.3	8.3	15.2	8.3	13.3	13.2
		FQ (%)	99.7	99.7	99.7	99.7	99.7	99.6
		SD (%)	0.3	0.5	0.2	0.5	0.14	0.3
	SDS	FS (min)	253.3	312.6	203.7	252.2	260.0	265.8
		SD (min)	7.6	13.3	8.7	13.3	21.8	17.1
		FQ (%)	98.8	98.5	98.2	98.5	98.2	97.8
		SD (%)	0.1	0.1	0.3	0.1	0.2	0.3
	TW80	FS (min)	71.8	139.4	83.2	139.4	118.3	111.7
		SD (min)	7.6	12.9	7.6	12.9	10.4	12.6
		FQ (%)	96.4	96.5	96.4	96.4	96.5	96.4
		SD (%)	0.3	1.0	0.9	0.3	0.9	0.9

Note: FS represents foam stability (min), FQ represents foam quality (%), and SD represents standard deviation (n = 3).

Table S5. Experimental design for batch experiment in the reactor for remediating TCE DNAPL contamination.

Group	Experiments	N_2/Foam flow rate (Q) (mL/min)			Concentration of AOS (mM) or AOS/SPS (mM/mM)		Experimental conditions	Measured parameters
		100	200	300	In generator	In reactor (after 2 h injection)		
Control	Only N_2 injection	a-1	a-2	a-3	-	-	$V_{reactor}$ = 1 L<br>$V_{pure\ TCE}$ = 7.5 mL<br>Operational time = 2 h	$TCE_{aq.}$<br>TCE_{gas}<br>TCE_{DNAPL}<br>SPS<br>Cl^-<br>pH<br>ORP<br>DO<br>SFT<br>CA
	Mixing w/ AOS surfactant	b-1	b-2	b-3	32	1.5<br>3.1<br>4.6		
	Mixing w/ SPS/AOS surfactant	c-1	c-2	c-3	32/50	1.5/2.4<br>3.1/4.8<br>4.6/7.2		
Foam injection	Regular foam	d-1	d-2	d-3	32	1.5<br>3.1<br>4.6		
	Oxidative foam (SPS 50 mM)	e-1	e-2	e-3	32/50	1.5/2.4<br>3.1/4.8<br>4.6/7.2		
	Oxidative foam (SPS 1700 mM)	f-1	f-2	f-3	32/1700	1.5/81.6<br>3.1/163.2<br>4.6/244.8		
	Oxidative foam (SPS 1700 mM)	g-1	g-2	g-3	32/1700	1.5/81.6<br>3.1/163.2<br>4.6/244.8	$V_{reactor}$ = 1 L<br>$V_{pure\ TCE}$ = 7.5 mL<br>Operational time = 240 h	

Note: V is volume, $V_{pure\ TCE}$ (mL) = 10 × (Solubility of TCE @25°C (mg/L) / TCE density (g/cm^3) × 10^{-3}). $Q_{N_2/Foam}$ (mL min^{-1}) is flowrate of N_2 injection for control experiment or foam injection. The experimental conditions of foam generator were fixed at screen #60 mesh and N_2/liquid ratio is 2.0/2.0 (L min^{-1})/L. V_{AOS} (mL) = Q_{Foam} (mL min^{-1}) × Time (min) × (100 - Foam quality (%)).

Table S6. Concentration of AOS and SPS with foam injection for 2 h at different foam flow rate.

Q_{Foam} (mL min^{-1})	$V_{liquid\ injected}$ [(1)] (mL)	C_{AOS} in foam generator (mM)	C_{AOS} in reactor [(2)] (mM)	C_{SPS} in foam generator [(3)] (mM)	C_{SPS} in reactor [(4)] (mM)
100	48	32	1.54	1700	81.60
200	96		3.07		163.20
300	144		4.61		244.80

Note: (1) $V_{liquid\ injected}$ is the volume of surfactant injected into the reactor.

$V_{liquid\ injectied}$ (mL) = Q_{Foam} (mL min^{-1}) × Time (min) × (100 – Foam quality (%))

(2) C_{AOS} in the reactor = $V_{liquid\ injected}$ (mL) × C_{AOS} in foam generator (mM) / $V_{reactor}$ (mL)

(3) C_{SPS} in foam generator (mM) was calculated basing on the theoretical molar ratio SPS/TCE (3/1) for completely degrading TCE in the reactor under 300 (mL min^{-1}) of foam flow rate. C_{SPS} in foam generator (mM) = ($Mass_{TCE\ DNAPL}$ (g) / MW_{TCE} (g/mol) × 3) × $V_{reactor}$ (mL) / $V_{liquid\ injected}$ (mL)

(4) C_{SPS} in reactor (mM) = C_{SPS} in the foam generator (mM) × $V_{liquid\ injected}$ (mL) / $V_{reactor}$ (mL)

Table S7. Comparison of prediction and oxidative foam injection experiment with 1700 mM of SPS after 240 h for TCE mineralization.

Q_{foam} (mL/min)	C_{PS} (1) (mM)	-d[TCE]/dt (2) (mM min^{-1})	TCE mass removal (mg)	Mineralization (%) (Theoretical calculation)	Mineralization (%) (Experiment)
100	81.6	2.33×10^{-3}	4408.7	40.3	39.7
200	163.2	4.07×10^{-3}	7701.1	70.3	66.2
300	244.8	5.62×10^{-3}	10633.9	97.1	73.8

Note: (1) Persulfate concentration (C_{PS}, mM) after foam injection for 2 hours = $V_{AOS\ injection}$ (mL) × C_{SPS} (mM) / $V_{reactor}$ where V_{AOS} (mL) = Q_{Foam} (mL min^{-1}) × Time (min) × (100 - Foam quality (%)), C_{SPS} = 1700 mM, and $V_{reactor}$ = 1000 mL. (2) The degradation rate of TCE by SPS at 25°C: -d[TCE]/dt (mM min^{-1}) = (6.90 × 10^{-5} $mM^{0.2}$ min^{-1}) $[TCE]^0[S_2O_8^{2-}]^{0.8}$.

Table S8. Reactions involved in the degradation of SPS in aqueous media.

Reaction
$S_2O_8^{2-} + H_2O \rightarrow 2HSO_4^{-} + ½\ O_2$
$HSO_4^{-} \rightarrow SO_4^{2-} + H^{+}$ (pK_a = 1.92)
$S_2O_8^{2-} + H_2O \rightarrow 2SO_4^{2-} + 2H^{+} + ½\ O_2$
$SO_4^{\bullet-} + H_2O \rightarrow SO_4^{2-} + H^{+} + {}^{\bullet}OH$

References

[1] R.A. Karjiban, M. Basri, M.B.A. Rahman, A.B. Salleh, Structural properties of nonionic Tween80 micelle in water elucidated by molecular dynamics simulation, Apcbee Proc. 3 (2012) 287-297. https://doi.org/10.1016/j.apcbee.2012.06.084

[2] S. Mandal, C. Banerjee, S. Ghosh, J. Kuchlyan, N. Sarkar, Modulation of the photophysical properties of curcumin in nonionic surfactant (Tween-20) forming micelles and niosomes: a comparative study of different microenvironments, J. Phys. Chem. B. 117 (2013) 6957-6968. https://doi.org/10.1021/jp403724g

[3] M.W. Sulek, T. Wasilewski, K.J. Kurzydłowski, The effect of concentration on lubricating properties of aqueous solutions of sodium lauryl sulfate and ethoxylated sodium lauryl sulfate, Tribol. Lett. 40 (2010) 337-345. https://doi.org/10.1007/s11249-010-9668-3

[4] L.R. Harutyunyan, R.S. Harutyunyan, Effect of amino acids on micellization and micellar parameters of anionic surfactant alpha olefin sulfonate C14–C16 in aqueous solutions: surface tension, conductometric, volumetric, and fluorescence studies, J. Chem. Eng. Data 64 (2019) 640-650. https://doi.org/10.1021/acs.jced.8b00886
